# Neurochemical Remodelling of the Enteric Nervous System Neurons in the Porcine Jejunum Following Low-Dose Glyphosate Exposure

**DOI:** 10.3390/ijms26209840

**Published:** 2025-10-10

**Authors:** Katarzyna Palus, Aleksandra Karpiesiuk, Barbara Jana

**Affiliations:** 1 Department of Clinical Physiology, Faculty of Veterinary Medicine, University of Warmia and Mazury in Olsztyn, Oczapowskiego 13, 10-718 Olsztyn, Poland; aleksandra.karpiesiuk@uwm.edu.pl; 2 Division of Reproductive Biology, Institute of Animal Reproduction and Food Research of the Polish, Academy of Sciences, Tuwima 10, 10-748 Olsztyn, Poland; b.jana@pan.olsztyn.pl

**Keywords:** glyphosate, enteric nervous system (ENS), neurochemical coding, immunofluorescence, jejunum, neurotoxicity, pig model

## Abstract

Glyphosate, a widely used herbicide, is under scrutiny for its potential neurotoxic effects. This study investigated whether oral exposure to glyphosate, even at doses currently considered safe in Europe, alters the neurochemical profile of enteric nervous system (ENS) neurons in the porcine jejunum. Fifteen immature female pigs were allocated to three groups: control (C), low-dose (G50; 50 µg/kg b.w./day), and higher-dose (G500; 500 µg/kg b.w./day). Following 28 days of exposure, jejunal samples were subjected to double-labelling immunofluorescence staining for neuronal markers, including Hu C/D and PACAP, CGRP, CART, nNOS, or VAChT. Results revealed dose-dependent neurochemical alterations across all enteric plexuses, with glyphosate increasing the number of neurons expressing PACAP, CGRP, CART, and nNOS, while reducing VAChT-positive neurons. The effect of glyphosate on enteric neurons appeared largely uniform across different plexus types, with more pronounced changes at the higher dose and only minor regional variation. Overall, the findings suggest that glyphosate exposure, even within regulatory limits, may alter the neurochemical profile of enteric neurons in a broadly uniform manner, potentially reflecting responses to oxidative stress or early neurotoxic effects, as reported in previous studies. This study challenges current safety thresholds and emphasises the need to reassess regulatory guidelines, particularly in the context of chronic exposure and potential risks to vulnerable populations.

## 1. Introduction

**Figure 1:**
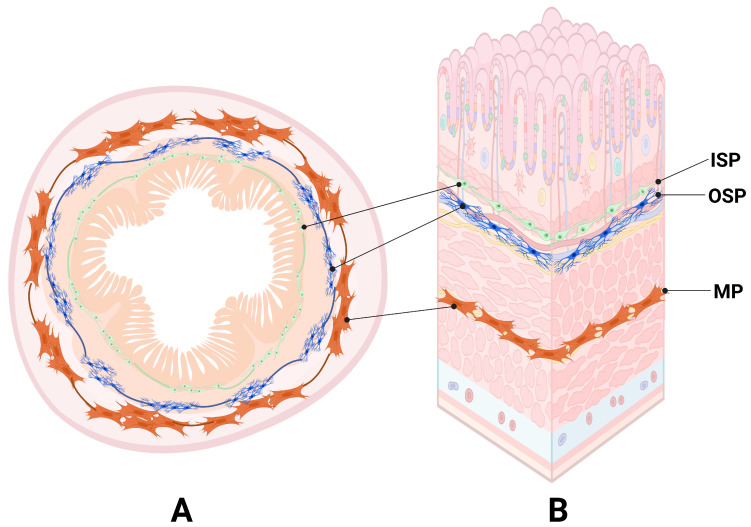
Anatomical (**A**) and histological (**B**) localisation of the myenteric (MP), outer submucosal (OSP), and inner submucosal (ISP) plexuses in the wall of the porcine jejunum. Created in BioRender. Palus, K. (2025) https://BioRender.com/4ril8ta. (accessed on 23 September 2025).

The enteric nervous system (ENS) constitutes a complex network of neurons and glial cells located within the wall of the gastrointestinal tract, responsible for the autonomous regulation of numerous digestive functions, ranging from peristalsis to the coordination of local immune responses [1,2]. In the small intestine of pigs, the ENS is organised into three main plexuses: the myenteric plexus (MP), situated between the longitudinal and circular muscle layers, primarily responsible for controlling intestinal motility via the regulation of smooth muscle contractions; the outer submucosal plexus (OSP), positioned on the inner side of the circular muscle layer; and the inner submucosal plexus (ISP), located adjacent to the outer muscularis layer of the mucosa. Both submucosal plexuses are involved in the regulation of secretion, absorption, and local blood flow [1,2,3] (Figure 1).

The ENS is characterised by high plasticity, defined as its capacity to undergo adaptive structural and functional changes in response to environmental stimuli, inflammatory processes, and tissue injury [4]. Key mechanisms of neuroplasticity include neurochemical remodelling (involving alterations in the expression of neurotransmitters and neuromodulators), neurogenesis, regenerative processes, and dynamic interactions with the gut microbiota and local immune system [4,5]. Environmental toxins and other exogenous factors have been shown to significantly affect the ENS, impacting its structure, physiological functions, and plasticity. Exposure to environmental contaminants, such as bisphenol A (BPA) [6], heavy metals (e.g., lead, arsenic, mercury) [7], pesticides [8], microplastics [9], and acrylamide present in foods [10], results in alterations in neurotransmitter immunoreactivity within the ENS.

To investigate these changes, the present study focused on selected markers with well-established roles in gut physiology. Pituitary adenylate cyclase-activating polypeptide (PACAP) regulates digestive juice secretion, hormone release, cellular proliferation, local ion transport, and induces dose-dependent relaxation of smooth muscle [11]. Neuronal nitric oxide synthase (nNOS) identifies nitric oxide-producing neurons, which mediate smooth muscle relaxation, inhibit neuromodulator release, and regulate gastrointestinal blood flow [12]. Vesicular acetylcholine transporter (VAChT) marks cholinergic neurons, which are crucial for excitatory neurotransmission, peristalsis, and stimulation of digestive secretions [13]. Calcitonin gene-related peptide (CGRP), a sensory neuropeptide, participates in nociceptive signalling, regulation of smooth muscle contractility, local blood flow, and modulation of other neurotransmitters [14]. Cocaine- and amphetamine-regulated transcript (CART), although less well understood in the ENS, has been reported to inhibit gastric secretion and promote colonic motility [15]. Together, these markers provide a comprehensive framework for assessing neurochemical alterations within the ENS, including those potentially induced by environmental factors such as glyphosate.

Glyphosate (*N*-(phosphonomethyl)glycine) is an organic phosphonate and the active ingredient in numerous commercially available herbicides, including the widely used formulation Roundup [16]. Glyphosate is primarily absorbed through the gastrointestinal tract, as well as via the skin and respiratory system, and exhibits limited metabolic capacity in mammals, with less than 1% of the administered dose being converted into aminomethylphosphonic acid (AMPA), its main metabolite. The majority of absorbed glyphosate is rapidly excreted in the urine unchanged, while approximately two-thirds of the orally administered dose is eliminated in the faeces as unabsorbed compound [17]. However, glyphosate has been shown to accumulate in the kidneys, liver, and gastrointestinal tract wall [18]. Despite its high efficacy in weed control, a growing body of evidence indicates potential health risks associated with glyphosate exposure [19]. Exposure to glyphosate or its commercial formulations has been reported to induce neurotoxicity in various animal species, including humans. Observed effects include, among others, disruptions in nerve cell development, impaired neurotransmission, oxidative stress, neuroinflammation, mitochondrial damage, and neuronal death [17,19,20]. Furthermore, glyphosate exhibits cytostatic and genotoxic effects, increases oxidative stress, adversely affects reproductive functions in animals, and may contribute to neoplasm development [21,22,23]. Exposure has also been shown to induce intestinal dysbiosis and damage to the intestinal microstructure, including microvilli, potentially resulting in increased intestinal barrier permeability and inflammatory responses [24,25,26]. Additionally, glyphosate disrupts lipid metabolism and alters liver functions, including the development of steatosis and bile acid metabolism disorders [24].

Although the neurotoxicity of glyphosate in the central nervous system and its effects on intestinal wall morphology has been relatively well characterised, knowledge regarding its impact on neurons within the enteric nervous system remains limited. Current evidence suggests that exposure to even low doses of glyphosate may modulate the neurochemical coding of enteric neurons and their receptor profiles in the small intestine [8,27]. Due to their physiological and anatomical similarity to humans—including comparable gastrointestinal tract structure, neuron density within nerve plexuses, and gut microbiota composition—pigs represent a valuable model for studying intestinal and nervous system functions [28]. The jejunum, as a segment of the small intestine with particularly high absorptive activity, constitutes a key site for interactions between environmental factors and gastrointestinal function [6].

In light of the above, the aim of this study was to investigate the effects of glyphosate on the distribution and immunoreactivity of selected neuroactive substances in enteric neurons of the porcine jejunum.

## 2. Results

### 2.1. The Myenteric Plexus (MP)

**Figure 2:**
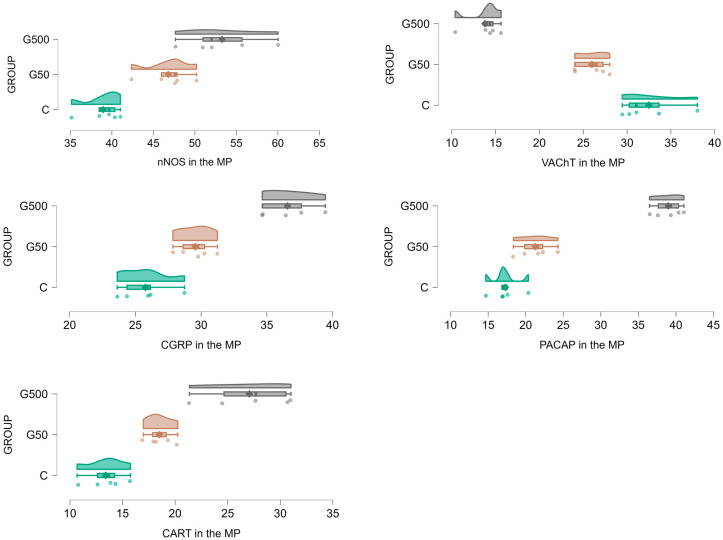
Raincloud plots showing the distribution of ENS neurons immunoreactive for nNOS, VAChT, CGRP, PACAP, and CART in the myenteric plexus (MP) of the jejunum in control (C) (green) and glyphosate-treated (G50 (brown), G500 (grey)) animals.

**Figure 3:**
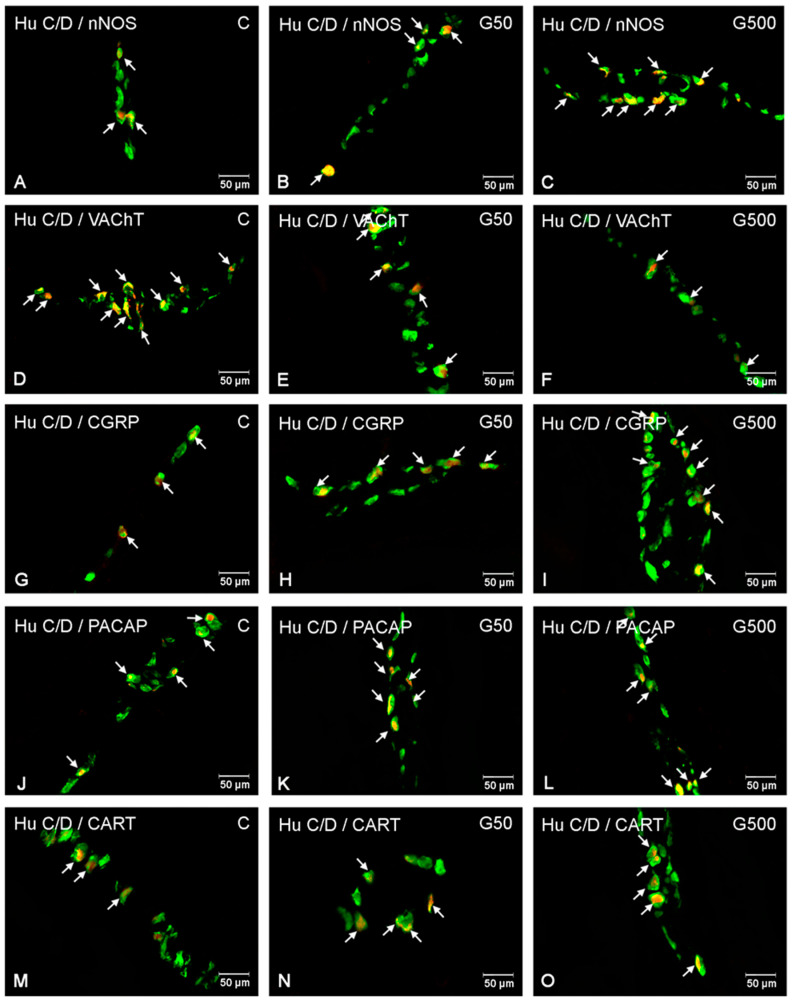
Representative photomicrographs showing enteric neurons located in the myenteric plexus (MP) of the porcine jejunum exhibiting immunoreactivity to the pan-neuronal marker Hu C/D (green) and one of the neurochemical markers: nNOS, VAChT, CGRP, PACAP, or CART (red). All images were acquired by merging the green and red fluorescence channels. (**A**) neurons immunoreactive to HuC/D and nNOS in the control group; (**B**) neurons immunoreactive to HuC/D and nNOS in the G50 group; (**C**) neurons immunoreactive to HuC/D and nNOS in the G500 group; (**D**) neurons immunoreactive to HuC/D and VAChT in the control group; (**E**) neurons immunoreactive to HuC/D and VAChT in the G50 group; (**F**) neurons immunoreactive to HuC/D and VAChT in the G500 group; (**G**) neurons immunoreactive to HuC/D and CGRP in the control group; (**H**) neurons immunoreactive to HuC/D and CGRP in the G50 group; (**I**) neurons immunoreactive to HuC/D and CGRP in the G500 group; (**J**) neurons immunoreactive to HuC/D and PACAP in the control group; (**K**) neurons immunoreactive to HuC/D and PACAP in the G50 group; (**L**) neurons immunoreactive to HuC/D and PACAP in the G500 group; (**M**) neurons immunoreactive to HuC/D and CART in the control group; (**N**) neurons immunoreactive to HuC/D and CART in the G50 group; (**O**) neurons immunoreactive to HuC/D and CART in the G500 group. Neurons immunoreactive to the respective neurochemical marker are indicated by arrows.

Glyphosate administration at both doses significantly increased the number of nNOS-immunoreactive (nNOS-IR) neurons in the MP (Figure 2 and Figure 3A–C). Statistically significant differences between groups were demonstrated (nNOS Model—F(2,12) = 2663.548, *p* < 0.000001; NOS Group—F(2,12) = 21.332, *p* = 0.00011). Post hoc analysis revealed significant differences between group G500 and group C (*p* = 0.00026) as well as group G500 and G50 (*p* = 0.031), and between group G50 and group C (*p* = 0.0099).

For VAChT-immunoreactive (VAChT-IR) neurons, glyphosate exposure induced a reduction in the neuronal population within the MP (Figure 2 and Figure 3D–F). Statistically significant differences between groups were observed (VAChT Model—F(2,12) = 1339.636, *p* < 0.000001; VAChT group—F(2,12) = 69.402, *p* < 0.000001). Post hoc comparisons indicated significant differences between group G500 and group C (*p* = 0.00019), group G500 and group G50 (*p* = 0.002), and group G50 and group C (*p* = 0.0045).

Regarding CGRP-immunoreactive (CGRP-IR) neurons, glyphosate treatment evoked a significant increase in their number (Figure 2 and Figure 3G–I). Significant differences between groups were found (CGRP Model—F(2,12) = 4286.763; *p* < 0.000001; CGRP Group—F(2,12) = 45.790, *p* = 0.000002). Post hoc analysis revealed differences between group G500 and group C (*p* = 0.00019), group G500 and G50 (*p* = 0.00031), and group G50 and group C (*p* = 0.016).

Similarly, for PACAP-immunoreactive (PACAP-IR) neurons, oral glyphosate exposure significantly increased their number within the MP (Figure 2 and Figure 3J–L). Statistically significant differences were observed between groups (PACAP Model—F(2,12) = 2326.925, *p* < 0.000001; PACAP Group—F(2,12) = 154.795, *p* < 0.000001). Post hoc analysis showed significant differences between group G500 and group C (*p* = 0.00019), group G500 and group G50 (*p* = 0.00019), and group G50 and group C (*p* = 0.025).

Glyphosate exposure at both tested doses also induced significant alterations in the population of CART-immunoreactive (CART-IR) neurons (Figure 2 and Figure 3M–O). Significant differences were confirmed between groups (CART Model—F(2,12) = 798.9123, *p* < 0.000001; CART Group—F(2,12) = 32.7982, *p* = 0.000014). Post hoc comparisons indicated differences between group G500 and group C (*p* = 0.00019), group G500 and group G50 (*p* = 0.00096), and group G50 and group C (*p* = 0.028).

### 2.2. The Outer Submucosal Plexus (OSP)

**Figure 4:**
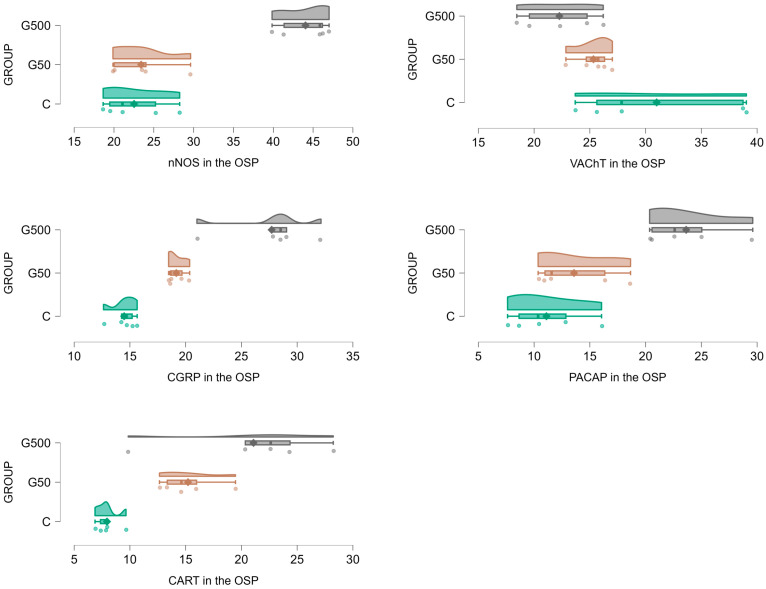
Raincloud plots showing the distribution of ENS neurons immunoreactive for nNOS, VAChT, CGRP, PACAP, and CART in the outer submucosal plexus (OSP) of the jejunum in control (C) (green) and glyphosate-treated (G50 (brown), G500 (grey)) animals.

**Figure 5:**
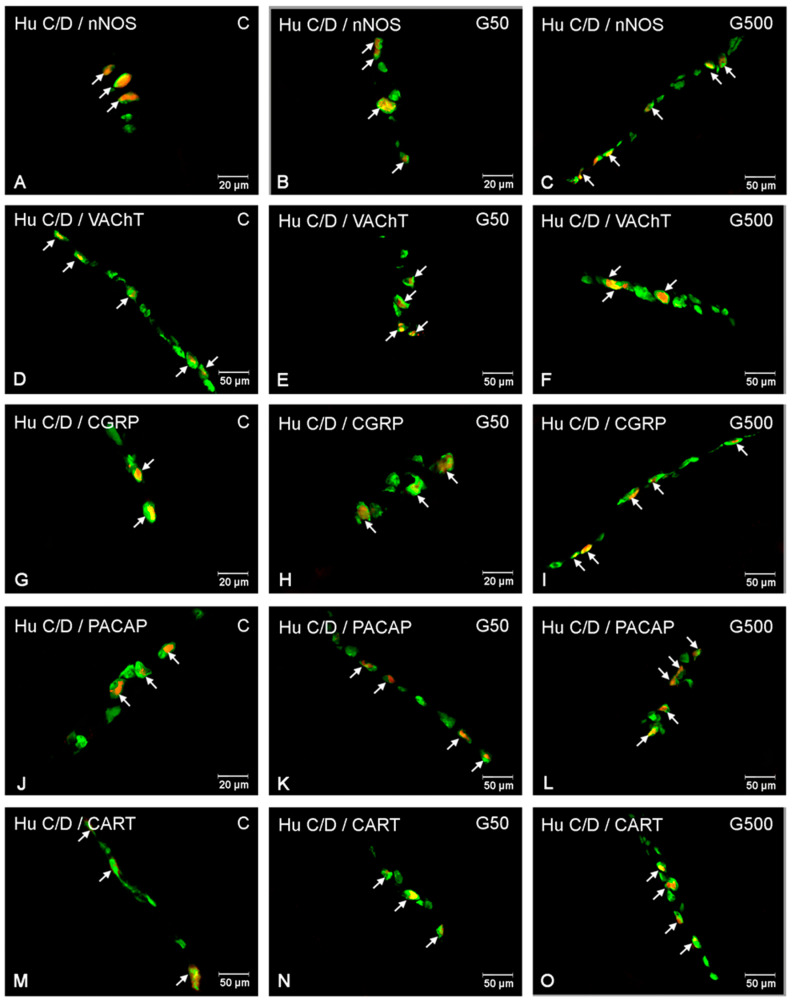
Representative photomicrographs showing enteric neurons located in the outer submucosal plexus (OSP) of the porcine jejunum exhibiting immunoreactivity to the pan-neuronal marker Hu C/D (green) and one of the neurochemical markers: nNOS, VAChT, CGRP, PACAP, or CART (red). All images were acquired by merging the green and red fluorescence channels. (**A**) neurons immunoreactive to HuC/D and nNOS in the control group; (**B**) neurons immunoreactive to HuC/D and nNOS in the G50 group; (**C**) neurons immunoreactive to HuC/D and nNOS in the G500 group; (**D**) neurons immu-noreactive to HuC/D and VAChT in the control group; (**E**) neurons immunoreactive to HuC/D and VAChT in the G50 group; (**F**) neurons immunoreactive to HuC/D and VAChT in the G500 group; (**G**) neurons immunoreactive to HuC/D and CGRP in the control group; (**H**) neurons immunoreactive to HuC/D and CGRP in the G50 group; (**I**) neurons immunoreactive to HuC/D and CGRP in the G500 group; (**J**) neurons immunoreactive to HuC/D and PACAP in the control group; (**K**) neurons im-munoreactive to HuC/D and PACAP in the G50 group; (**L**) neurons immunoreactive to HuC/D and PACAP in the G500 group; (**M**) neurons immunoreactive to HuC/D and CART in the control group; (**N**) neurons immunoreactive to HuC/D and CART in the G50 group; (**O**) neurons immunoreactive to HuC/D and CART in the G500 group. Neurons immunoreactive to the respective neurochemical marker are indicated by arrows.

Within the OSP, supplementation with the higher dose of glyphosate resulted in an increase in the number of nNOS-IR neurons (Figure 4 and Figure 5A–C). Statistically significant differences between groups were demonstrated (nNOS Model—F(2,12) = 947.6607, *p* < 0.000001; NOS Group—F(2,12) = 52.0689, *p* = 0.000001). Post hoc analysis revealed significant differences between group G500 and group C (*p* = 0.00019), as well as between group G500 and G50 (*p* = 0.00019).

For VAChT-IR neurons, glyphosate exposure induced a reduction in the neuronal population within the OSP (Figure 4 and Figure 5D–F). Statistically significant differences were observed between groups (VAChT Model—F(2,12) = 456.1308, *p* < 0.000001; VAChT group—F(2,12) = 4.3402, *p* = 0.038). Post hoc comparisons indicated significant differences between group G500 and group C (*p* = 0.033).

Regarding CGRP-IR neurons, glyphosate treatment evoked a significant increase in their number (Figure 4 and Figure 5G–I). Statistically significant differences between groups were confirmed (CGRP Model—F(2,12) = 1002.755; *p* < 0.000001; CGRP Group—F(2,12) = 35.966, *p* = 0.000009). Post hoc analysis revealed differences between group G500 and group C (*p* = 0.00019), group G500 and group G50 (*p* = 0.00059), and group G50 and group C (*p* = 0.03).

Similarly, for PACAP-IR neurons, oral glyphosate exposure significantly increased their number within the OSP (Figure 4 and Figure 5J–L). Statistically significant differences between groups were observed (PACAP Model—F(2,12) = 292.8402, *p* < 0.000001; PACAP Group—F(2,12) = 16.5784, *p* = 0.00035). Post hoc analysis showed significant differences between group G500 and group C (*p* = 0.00057), and between group G500 and group G50 (*p* = 0.0026).

Glyphosate at the higher dose also induced significant alterations in the population of CART-IR neurons within the OSP (Figure 4 and Figure 5M–O). Statistically significant differences between groups were demonstrated (CART Model—F(2,12) = 174.3227, *p* < 0.000001; CART Group—F(2,12) = 11.5788, *p* = 0.0016). Post hoc comparisons indicated significant differences between group G500 and group C (*p* = 0.0013).

### 2.3. The Inner Submucosal Plexus (ISP)

**Figure 6:**
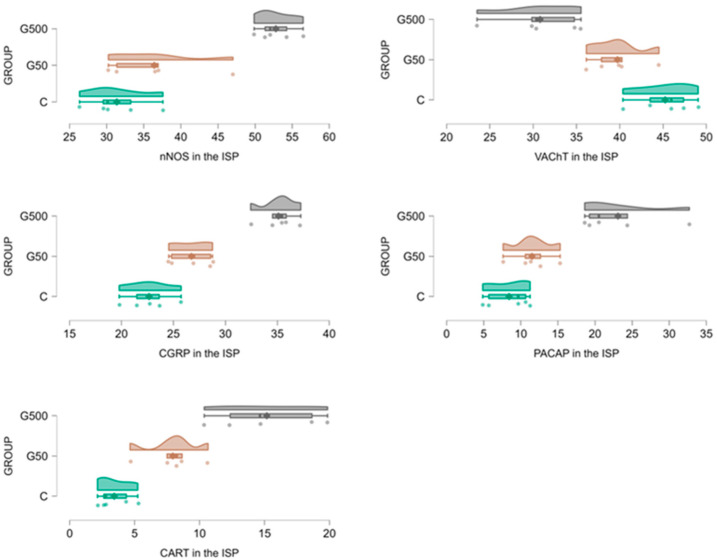
Raincloud plots showing the distribution of ENS neurons immunoreactive for nNOS, VAChT, CGRP, PACAP, and CART in the inner submucosal plexus (ISP) of the jejunum in control (C) (green) and glyphosate-treated (G50 (brown), G500 (grey)) animals.

**Figure 7:**
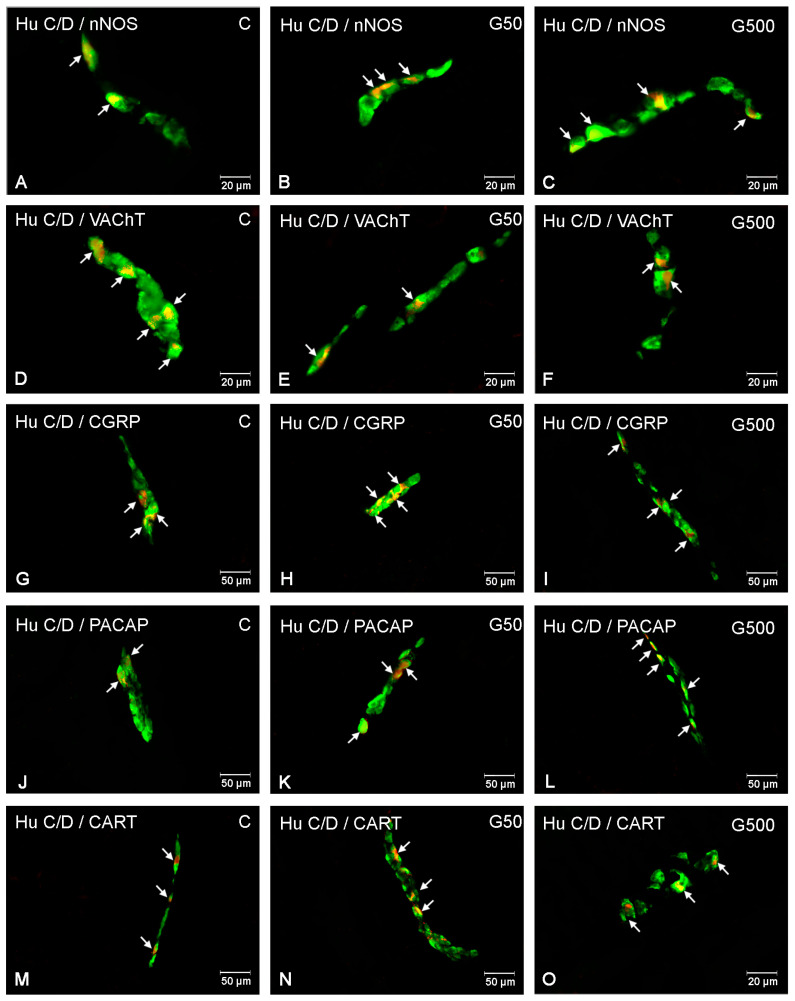
Representative photomicrographs showing enteric neurons located in the inner submucosal plexus (OSP) of the porcine jejunum exhibiting immunoreactivity to the pan-neuronal marker Hu C/D (green) and one of the neurochemical markers: nNOS, VAChT, CGRP, PACAP, or CART (red). All images were acquired by merging the green and red fluorescence channels. (**A**) neurons immunoreactive to HuC/D and nNOS in the control group; (**B**) neurons immunoreactive to HuC/D and nNOS in the G50 group; (**C**) neurons immunoreactive to HuC/D and nNOS in the G500 group; (**D**) neurons immu-noreactive to HuC/D and VAChT in the control group; (**E**) neurons immunoreactive to HuC/D and VAChT in the G50 group; (**F**) neurons immunoreactive to HuC/D and VAChT in the G500 group; (**G**) neurons immunoreactive to HuC/D and CGRP in the control group; (**H**) neurons immunoreactive to HuC/D and CGRP in the G50 group; (**I**) neurons immunoreactive to HuC/D and CGRP in the G500 group; (**J**) neurons immunoreactive to HuC/D and PACAP in the control group; (**K**) neurons im-munoreactive to HuC/D and PACAP in the G50 group; (**L**) neurons immunoreactive to HuC/D and PACAP in the G500 group; (**M**) neurons immunoreactive to HuC/D and CART in the control group; (**N**) neurons immunoreactive to HuC/D and CART in the G50 group; (**O**) neurons immunoreactive to HuC/D and CART in the G500 group. Neurons immunoreactive to the respective neurochemical marker are indicated by arrows. Additionally, two-way ANOVA analyses showed strong and highly significant main effects of glyphosate exposure and plexus type across all examined neuronal markers (nNOS, VAChT, CGRP, PACAP, and CART) (Supplementary Tables S2–S11). Importantly, F-values for the interaction between experimental group and plexus type were consistently much smaller than those for the main effects (Supplementary Tables S2, S4, S6, S8 and S10). This disparity supports the conclusion that low-dose glyphosate exposure exerts a largely uniform effect on enteric neurons, irrespective of plexus type.

In the ISP, administration of the higher glyphosate dose induced an increase in the number of nNOS-IR neurons (Figure 6 and Figure 7A–C). Statistically significant differences between groups were demonstrated (nNOS Model—F(2,12) = 1056.790, *p* < 0.000001; nNOS Group—F(2,12) = 27.356, *p* = 0.000034). Post hoc analysis revealed significant differences between group G500 and group C (*p* = 0.00022), as well as between group G500 and G50 (*p* = 0.00059).

For VAChT-IR neurons, glyphosate exposure led to a reduction in the neuronal population within the ISP (Figure 6 and Figure 7D–F). Statistically significant differences between groups were observed (VAChT Model—F(2,12) = 1507.773, *p* < 0.000001; VAChT Group—F(2,12) = 17.934, *p* = 0.00025). Post hoc comparisons indicated significant differences between group G500 and group C (*p* = 0.00035) and between group G500 and G50 (*p* = 0.0085).

Regarding CGRP-IR neurons, glyphosate treatment evoked a significant increase in their number (Figure 6 and Figure 7G–I). Statistically significant differences between groups were confirmed (CGRP Model—F(2,12) = 2924.056, *p* < 0.000001; CGRP Group—F(2,12) = 49.479, *p* = 0.000002). Post hoc analysis showed significant differences between group G500 and group C (*p* = 0.00019), group G500 and G50 (*p* = 0.00025), and group G50 and group C (*p* = 0.02).

Similarly, for PACAP-IR neurons, oral glyphosate exposure significantly increased their number within the ISP (Figure 6 and Figure 7J–L). Statistically significant differences between groups were demonstrated (PACAP Model—F(2,12) = 183.6521, *p* < 0.000001; PACAP Group—F(2,12) = 17.7963, *p* = 0.00026). Post hoc comparisons revealed significant differences between group G500 and group C (*p* = 0.00045) and between group G500 and G50 (*p* = 0.0022).

Glyphosate at the higher dose also induced significant alterations in the population of CART-IR neurons within the ISP (Figure 6 and Figure 7M–O). Statistically significant differences between groups were confirmed (CART Model—F(2,12) = 154.6074, *p* < 0.000001; CART Group—F(2,12) = 23.1052, *p* = 0.000077). Post hoc analysis indicated significant differences between group G500 and group C (*p* = 0.00024) and between group G500 and G50 (*p* = 0.0037).

## 3. Discussion

The present study demonstrated that oral exposure to glyphosate, at doses corresponding to both the TMDI and ADI, significantly alters the neurochemical phenotype of intestinal neurons in the jejunum of young gilts. An increase was observed in the number of neurons immunoreactive for neuroactive substances with documented neuromodulatory and neuroprotective effects (PACAP, CGRP, CART, and nNOS), accompanied by a decrease in the population of VAChT-immunoreactive neurons. These findings, consistent with previous studies on the effects of environmental toxins on the ENS, underscore the high neuroplasticity of the enteric nervous system in adapting to disruptions of gastrointestinal homeostasis [1,2,3,4,6,7,8].

The observed changes appear to result from the neurotoxic effects of glyphosate on intramural neurons of the ENS. Literature evidence indicates that glyphosate exerts multifaceted effects on the nervous system, leading to disruptions in neurotransmission and synaptic function, as well as an enhanced inflammatory response. These effects may, in turn, contribute to the development of neurodegenerative diseases, depressive symptoms, anxiety disorders, and cognitive dysfunction [19,20,29,30,31,32]. A key mechanism underlying glyphosate-induced neurotoxicity involves the activation of chronic inflammation via Toll-like receptor 4 (TLR4), which triggers the release of pro-inflammatory cytokines (TNF-α, IL-1β) and the generation of reactive oxygen species (ROS) [19]. Oxidative stress, a central component of glyphosate cytotoxicity, manifests as increased ROS production, leading to lipid peroxidation, protein oxidation, and DNA damage [19,33]. Glyphosate-induced cytotoxicity are further exacerbated by inhibition of endogenous antioxidant defences, including reduced enzymatic activity of superoxide dismutase (SOD), catalase (CAT), and glutathione peroxidase (GPx), decreased glutathione (GSH) levels, and dysregulation of Nrf2-dependent gene expression [34,35]. Elevated levels of malondialdehyde (MDA), apoptosis markers (e.g., Bax, caspase-3/9), and NF-κB activation confirm the induction of inflammation, apoptosis, and tissue damage [35].

Oxidative stress is also associated with numerous morphological changes in the intestines observed during glyphosate exposure [34,35,36,37]. An experiment conducted on rats, which were administered glyphosate (5–500 mg/kg) for 35 days, showed a decrease in the villus height-to-crypt depth ratio in both the duodenum and jejunum. In addition, reduced activity of antioxidant enzymes (T-SOD, GSH-Px) as well as elevated malondialdehyde (MDA) levels were observed, indicative of oxidative stress. Furthermore, glyphosate increased the expression of genes associated with the inflammatory response (IL-1β, IL-6, TNF-α) [34]. A study conducted on C57BL/6 mice showed that perinatal exposure to glyphosate (0.5% in water) altered the morphometry of the jejunum, increasing the proportion of intraepithelial lymphocytes and goblet cells, and modifying the collagen fibre content. Hypertrophy of the muscular and submucosal layers, as well as changes in the number of neurons in intestinal nerve plexuses, were also observed [36]. Experiments on zebrafish embryos demonstrated that exposure to glyphosate (5–50 μg/mL) resulted in decreased levels of acetylated α-tubulin and a reduction in the proportion of polymeric tubulin, suggesting effects on microtubule stability and potential cytoskeletal damage [37]. Another study using this model confirmed that exposure to glyphosate (3.5 mg/L) for 21 days decreased mRNA levels of genes associated with tight junctions (claudin-5, occludin, ZO-1) and altered diamine oxidase and D-lactose levels, indicating increased intestinal permeability. In addition, elevated IL-1β and IL-8 levels, alongside decreased IL-10 and TGF-β levels, suggested inflammation within the intestines [35]. In a study carried out on piglets, a diet containing glyphosate (10–40 mg/kg) for 35 days had no observable effect on overall small intestinal morphology. However, increased activity of antioxidant enzymes (CAT, SOD) and changes in the expression of genes associated with oxidative stress and inflammatory responses were observed in the duodenum [26]. Previous work by the authors demonstrated that glyphosate exposure at TMDI and ADI doses altered the expression of SOD-encoding mRNA within the small intestine of pigs [8]. Although no structural changes were noted during this experiment, in which low doses of glyphosate were used, the observed neurochemical alterations in ENS neurons can be interpreted as a manifestation of functional adaptation and neuroplasticity.

It is noteworthy that the neuroactive substances showing increased immunoreactivity in this study have well-documented neuroprotective and neuromodulatory functions. PACAP inhibits apoptosis and oxidative stress through activation of the cAMP/PKA and PI3K/AKT pathways, and exerts anti-inflammatory effects by suppressing the production of pro-inflammatory cytokines (e.g., TNF-α, IL-6) while promoting the secretion of anti-inflammatory cytokines (e.g., IL-10) [11,38]. CART exerts neuroprotective effects by modulating neurogenesis, neuronal reorganisation, and inflammatory responses [15,39]. Nitric oxide (NO), synthesised by nNOS, primarily acts as an inhibitory mediator in the enteric nervous system, modulating gastrointestinal motility, mediating synaptic neuroplasticity, and regulating immune responses via NO-dependent effects on macrophages and microglia [40,41]. In contrast, CGRP is involved in neuromodulation, pain transmission, and regulation of intestinal motility, exhibiting anti-inflammatory properties through modulation of macrophage and dendritic cell activity [14,42]. Moreover, increased immunoreactivity of PACAP, CART, nNOS, and CGRP in ENS neurons has been documented during pathological and toxic processes in the gastrointestinal tract [3,6,9,14,15,43,44], suggesting their participation in adaptive and/or defensive responses of ENS neurons to glyphosate-induced disturbances of small intestinal homeostasis. However, the reduction in the number of cholinergic neurons observed in the present study following glyphosate exposure may result from disruptions in enzymatic pathways within ENS neurons. Glyphosate intoxication has been shown to disrupt the functioning of adrenergic, dopaminergic, and serotonergic systems, and to inhibit the activity of numerous enzymes involved in neurotransmitter release, including acetylcholinesterase (AChE) [19,45]. Furthermore, a reduction in the population of VAChT-immunoreactive neurons has been reported in various pathological conditions affecting the gastrointestinal tract [27,46], suggesting that acetylcholine synthesis may be inhibited in ENS neurons, while the synthesis of other neuroactive substances involved in neuroprotective processes may be upregulated.

An increasing number of studies highlight the significance of the microbiota–gut–brain axis, in which intestinal microorganisms play a crucial role in regulating ENS function by modulating the expression of neuromodulators, the production of short-chain fatty acids (SCFAs), cytokines, and tryptophan metabolites [47,48]. Numerous in vivo and in vitro studies indicate that glyphosate, at varying doses, can induce dysbiosis characterised by reductions in *Corynebacterium*, *Firmicutes*, *Bacteroidetes*, and *Lactobacillus* levels, alongside an increase in the abundance of potentially pathogenic bacteria [25,26,34,35,49]. In this context, the neurochemical alterations observed in the present study may be secondary to glyphosate-induced dysbiosis, a factor that warrants further investigation to elucidate the mechanisms underlying glyphosate’s toxic effects on the ENS.

Despite the strengths of this study, several limitations should be acknowledged. First, only low, environmentally relevant doses of glyphosate were investigated, while high toxic concentrations were not included, precluding direct comparisons between low and high exposures. Second, nuclear counterstaining (e.g., DAPI) was not performed, as the neuroactive substances examined are localised within the cytoplasm and neuronal processes. Nonetheless, the absence of nuclear labelling may reduce the precision of neuronal identification and complicate the interpretation of immunohistochemical findings. As a future perspective, it would be valuable to broaden the comparative framework to include species that are phylogenetically close to pigs but adapted to different ecosystems. For example, Bombardi et al. [50] described nitrergic and substance P neurons in the intestine of the bottlenose dolphin (*Tursiops truncatus*), a marine member of the Cetartiodactyla. This provides a baseline reference for ENS organisation in a clade closely related to Suidae. Although dolphins are not directly exposed to agricultural environments, glyphosate residues have been reported in coastal and marine waters, raising the possibility of indirect exposure [51,52]. Comparative studies across terrestrial and marine Cetartiodactyla would therefore allow distinctions to be drawn between evolutionary determinants and environmental or ecotoxicological drivers of ENS neurochemical remodelling. Such an approach could substantially expand our understanding of how environmental contaminants interact with conserved neurochemical pathways across mammals.

## 4. Materials and Methods

The experimental procedures, including euthanasia of the animals, were carried out in accordance with applicable regulations regarding animal protection and welfare, both at EU and national level (Legislative Decree 26/2014 implementing EU Directive 2010/63/EU), and were approved by the Local Committee for Animal Experiments in Olsztyn (Approval No. 62/2020, approval date 21 October 2020).

### 4.1. Animals and Treatment

**Figure 8:**
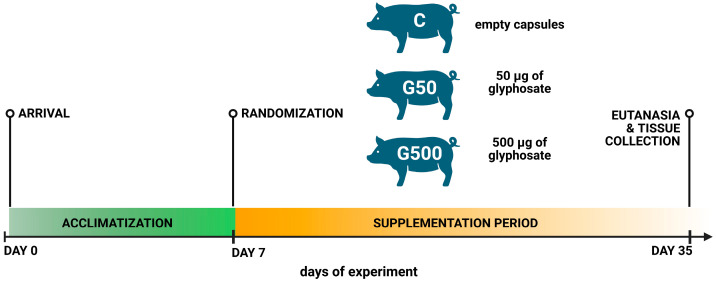
The experimental scheme timeline showing the main phases of the study. Green indicates a 7-day acclimatization period; orange indicates a 28-day supplementation period. Created in BioRender. Palus, K. (2025) https:/BioRender.com/5mg9h9w. (accessed on 23 September 2025).

The study was conducted on 15 sexually immature gilts (*Sus scrofa domestica*) of the Danish Landrace breed, approximately 8 weeks old and weighing around 20 kg. The animals were obtained from a local commercial pig farm (Sławomir Melibruda Pig Farm, Bryski, Rościszewo, Poland). Following a 1 week acclimatisation period, pigs were randomly assigned to one of three experimental groups (five animals per group): control group (C), experimental group 1 (G50), and experimental group 2 (G500). Group C received empty gelatine capsules, whereas group G50 received a low dose of glyphosate ((analytical standard, purity > 99.5%, Sigma-Aldrich, St. Louis, MO, USA; CAS No. 1071-83-6)), corresponding to the theoretical maximum daily intake (TMDI) in Europe, namely 50 µg/kg body weight per day. Group G500 received a higher dose of glyphosate, corresponding to the acceptable daily intake (ADI) of 500 µg/kg body weight per day. The animals were fed twice daily with commercially available feed adapted to this species and had constant access to water (according to the experimental scheme presented in Figure 8).

### 4.2. Tissue Sampling

**Figure 9:**
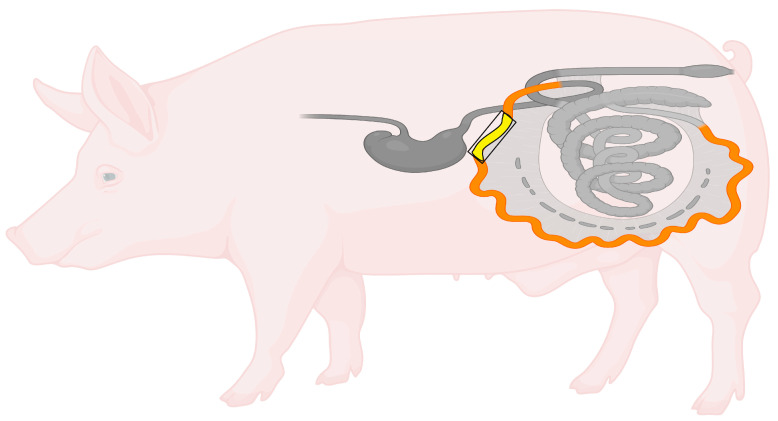
The anatomical scheme illustrating the location of the jejunum in the gastrointestinal tract of the pig, with the sampling site indicated. Created in BioRender. Palus, K. (2025) https://BioRender.com/04oynz0. (accessed on 23 September 2025).

The pigs were euthanised after 28 days of the experiment by administering a lethal dose of sodium pentobarbital (Morbital (Biowet Puławy, Puławy, Poland; 0.6 mL/kg body weight, i.v.); 0.6 mL/kg body weight, i.v.), preceded by premedication with azaperone (Stresnil, Janssen Pharmaceutica N.V., Beerse, Belgium.; 4 mg/kg body weight, i.m.). Subsequently, 2 cm long fragments of the jejunum, located approximately 40 cm distal to the pylorus (as shown in Figure 9), were collected from all animals and fixed by immersion in 4% buffered paraformaldehyde solution (pH 7.4) for 1 h. Thereafter, the fixed fragments were transferred to a phosphate-buffered saline solution (PBS, pH 7.4), which was replaced three times at 24 h intervals, and finally placed in a 30% sucrose solution at 4 °C for a minimum of three weeks.

### 4.3. Cutting and Immunofluorescence Staining

The jejunal fragments obtained from each pig and prepared as described above were used to create small freezing blocks, which were cut into serial 12 µm thick full-thickness transverse sections using a Microm HM 560 cryostat (Carl Zeiss, Oberkochen, Germany). The sections were then subjected to double immunofluorescence staining, following the procedure previously described by Palus et al. [53]. Initially, the sections were rinsed in 0.1 M PBS (3 × 10 min) and then blocked in a solution containing 10% horse serum, 10% goat serum, 0.1% bovine serum albumin in 0.1 M PBS, 1% Triton X-100, 0.05% thiomersal, and 0.01% sodium azide for 1 h. The sections were subsequently incubated with a mixture of primary antibodies against Hu C/D (pan-neuronal marker), pituitary adenylate cyclase-activating polypeptide (PACAP), calcitonin gene-related peptide (CGRP), cocaine- and amphetamine-regulated transcript (CART), neuronal nitric oxide synthase (nNOS), and vesicular acetylcholine transporter (VAChT) (antibody specifications are provided in Table 1) for 24 h. On the following day, the sections were rinsed three times in 0.1 M PBS (3 × 10 min) and incubated with a mixture of species-specific secondary antibodies (Table 1). To validate the staining, specificity tests were performed, including substitution and pre-absorption controls for primary antibodies and omission controls for secondary antibodies.

### 4.4. Microscopic Analysis and Counting

The sections were observed using an Olympus BX51 microscope (Olympus, Tokyo, Japan) coupled with a camera (Olympus XM 10) and a computer with appropriate software (Olympus Cell F, Olympus, Tokyo, Japan). The number of immunopositive neurons, corresponding to the neuroactive substances under study (PACAP, CGRP, CART, nNOS, VAChT), was determined relative to Hu C/D-positive neurons (pan-neuronal marker) and expressed as a percentage. For each intestinal plexus type (MP, OSP and ISP), at least 500 Hu C/D-positive cells were counted per pig. The neurons were counted when their nuclei were clearly visible. In order to avoid double-counting the same neurons, sections separated by at least 100 μm were selected for analysis. It should be noted that within each examined plexus, individual neurons may synthesise more than one neuroactive substance, and therefore neurochemical markers can co-localise within the same neuronal cell bodies, which explains why the percentage sums of neuronal populations in a given plexus may exceed 100%.

### 4.5. Statistical Analysis

Quantitative data were collected and presented as a mean ± standard deviation (SD). Prior to parametric testing, the assumptions of normality and homogeneity of variances were examined. Normality of residuals was evaluated with the Shapiro–Wilk test, and homogeneity of variances with Levene’s test. Both assumptions were met (*p* > 0.05). In order to detect differences between the groups, a one-way analysis of variance (ANOVA) with Tukey’s HSD post hoc test was used. The analyses were conducted using the Statistica version 13.3 software (TIBCO Software Inc., Palo Alto, CA, USA). A *p*-value < 0.05 was considered statistically significant. Detailed descriptive statistics and two-way analysis of variance (ANOVA) results are provided in the Supplementary Materials.

## 5. Conclusions

In summary, oral exposure to glyphosate at low, legally permitted doses induces alterations in the neurochemical profile of enteric neurons in the porcine jejunum. The changes were particularly pronounced in animals receiving the higher glyphosate dose, indicating a dose-dependent escalation of its detrimental effects. The effect appeared largely uniform across all enteric plexuses, regardless of plexus type. Accumulating evidence regarding glyphosate cytotoxicity indicates that the observed changes may result from its neurotoxic action, mediated by oxidative stress or direct harmful effects on ENS neurons. Although the glyphosate doses employed in the present study are consistent with current safety standards (TMDI, ADI), the findings suggest that even low-level, chronic exposure can elicit neurochemical alterations in the ENS. This challenges previous assumptions that such exposure levels are entirely safe from a public health perspective. Given the growing body of evidence highlighting the neurotoxic and pro-inflammatory potential of glyphosate, a re-evaluation of acceptable exposure limits is warranted, particularly for vulnerable populations, including children, pregnant women, and individuals with intestinal disorders. Simultaneously, toxicological assessments should also consider effects on the gut microbiota, mucosal immunity, and the functioning of the gut–brain axis.

## Figures and Tables

**Table 1:** Details of antibodies used in the present study.

**Primary antibody**				
**Antigen**	**Host species**	**Cat No.**	**Dilution**	**Supplier**
Hu C/D	mouse	A-21271	1:1000	Thermo Fisher Scientific, Waltham, MA, USA
PACAP	guinea pig	T-5039	1:3000	Peninsula, San Carlos, CA, USA
CGRP	rabbit	MAB317	1:4000	Millipore, Burlington, MA, USA
CART	rabbit	H-003-61	1:8000	Phoenix Pharmaceuticals, Burlingame, CA, USA
nNOS	rabbit	AB5380	1:2000	Sigma-Aldrich, Saint Louis, MO, USA
VAChT	rabbit	H-V007	1:2000	Phoenix Pharmaceuticals, Burlingame, CA, USA
**Secondary antibodies**				
**Reagents**	**Cat No.**	**Dilution**	**Supplier**	
Alexa Fluor 488 donkey anti- mouse IgG	A21202	1:1000	Thermo Fisher Scientific, Waltham, MA, USA	
Alexa Fluor 546 donkey anti- guinea pig IgG	A11074	1:1000	Thermo Fisher Scientific, Waltham, MA, USA	
Alexa Fluor 546 goat anti- rabbit IgG	A11010	1:1000	Thermo Fisher Scientific, Waltham, MA, USA	

## Data Availability

All relevant data are contained within the manuscript.

## Supplementary Materials

The following supporting information can be downloaded at: https://www.mdpi.com/article/10.3390/ijms26209840/s1.
