# Supplementary material for: Neurochemical Remodelling of the Enteric Nervous System Neurons in the Porcine Jejunum Following Low-Dose Glyphosate Exposure
Source: Int J Mol Sci. 2025 Oct 10;26(20):9840. doi: 10.3390/ijms26209840 (PMC12564263; doi:10.3390/ijms26209840)
Supplement: Supplementary file 1 [file ijms-26-09840-s001.zip › ijms-3873345-supplementary.pdf]

Supplementary Table S1. Descriptive statistics of PACAP-, CGRP-, CART-, nNOS- and VAChT-immunoreactive neurons in the myenteric (MP), outer submucosal (OSP), and inner submucosal (ISP) plexuses of the porcine jejunum in control and glyphosate-treated groups (G50, G500).

Variable	Valid N	Mean	Median	Minimum	Maximum	Lower Quartile (Q1)	Upper Quartile (Q3)	Standard Deviation (SD)	95% CI Lower Bound for SD	95% CI Upper Bound for SD
**Control group**										
**PACAP MP**	5	17.28	16.97	14.68	20.36	16.84	17.54	2.04	1.22	5.85
**PACAP OSP**	5	11.11	10.36	7.62	16.05	8.65	12.85	3.40	2.04	9.77
**PACAP ISP**	5	8.42	9.65	4.87	11.25	5.69	10.65	2.94	1.76	8.45
**CGRP MP**	5	25.76	25.96	23.62	28.74	24.36	26.14	1.98	1.18	5.68
**CGRP OSP**	5	14.48	14.69	12.63	15.65	14.25	15.20	1.16	0.70	3.34
**CGRP ISP**	5	22.66	22.65	19.78	25.74	21.48	23.63	2.24	1.34	6.44
**CART MP**	5	13.40	13.68	10.68	15.75	12.63	14.25	1.89	1.13	5.44
**CART OSP**	5	7.94	7.86	6.87	9.65	7.36	7.98	1.05	0.63	3.02
**CART ISP**	5	3.43	2.78	2.14	5.24	2.63	4.36	1.31	0.79	3.77
**nNOS MP**	5	38.93	39.67	35.14	41.02	38.47	40.36	2.32	1.39	6.67
**nNOS OSP**	5	22.53	21.06	18.64	28.25	19.47	25.21	4.08	2.44	11.72
**nNOS ISP**	5	31.37	30.14	26.35	37.58	29.54	33.25	4.25	2.54	12.21
**VACHT MP**	5	32.48	31.05	29.45	38.02	30.25	33.64	3.47	2.08	9.98
**VACHT OSP**	5	30.99	27.85	23.69	39.04	25.63	38.74	7.36	4.41	21.15
**VACHT ISP**	5	45.25	45.95	40.36	49.05	43.51	47.36	3.40	2.04	9.78
**G50 group**										
**PACAP MP**	5	21.28	21.63	18.36	24.30	19.87	22.25	2.27	1.36	6.54
**PACAP OSP**	5	13.58	11.57	10.36	18.65	10.98	16.34	3.69	2.21	10.61
**PACAP ISP**	5	11.51	11.36	7.62	15.30	10.62	12.65	2.81	1.68	8.08
**CGRP MP**	5	29.55	29.78	27.85	31.24	28.64	30.25	1.33	0.80	3.83
**CGRP OSP**	5	19.16	18.65	18.47	20.36	18.65	19.67	0.82	0.49	2.36

**CGRP ISP**	5	26.74	26.98	24.55	28.76	24.82	28.57	2.00	1.20	5.74
**CART MP**	5	18.51	18.32	16.98	20.25	17.86	19.15	1.25	0.75	3.59
**CART OSP**	5	15.21	14.62	12.65	19.47	13.35	15.98	2.70	1.62	7.75
**CART ISP**	5	7.94	8.25	4.65	10.65	7.50	8.65	2.18	1.30	6.26
**nNOS MP**	5	46.80	47.61	42.36	50.21	45.98	47.85	2.91	1.74	8.35
**nNOS OSP**	5	23.41	23.54	19.87	29.62	20.02	24.01	3.97	2.38	11.40
**nNOS ISP**	5	36.42	36.51	30.24	47.02	31.38	36.94	6.64	3.98	19.07
**VACHT MP**	5	25.97	26.51	24.03	28.02	24.06	27.25	1.84	1.10	5.29
**VACHT OSP**	5	25.32	25.75	22.85	27.02	24.68	26.32	1.63	0.97	4.67
**VACHT ISP**	5	39.73	39.85	36.14	44.52	37.89	40.25	3.14	1.88	9.03
					**G500 group**					
**PACAP MP**	5	38.98	39.33	36.48	41.06	37.65	40.36	1.90	1.14	5.45
**PACAP OSP**	5	23.64	22.61	20.36	29.61	20.58	25.04	3.83	2.30	11.02
**PACAP ISP**	5	23.09	20.52	18.61	32.70	19.25	24.36	5.82	3.49	16.72
**CGRP MP**	5	36.57	36.45	34.65	39.45	34.68	37.62	2.04	1.22	5.87
**CGRP OSP**	5	27.71	28.52	21.01	32.14	27.85	29.05	4.09	2.45	11.76
**CGRP ISP**	5	35.10	35.45	32.43	37.25	34.54	35.85	1.79	1.07	5.13
**CART MP**	5	27.05	27.68	21.36	31.02	24.65	30.54	4.08	2.44	11.71
**CART OSP**	5	21.09	22.63	9.85	28.24	20.35	24.36	6.91	4.14	19.86
**CART ISP**	5	15.17	14.65	10.36	19.85	12.36	18.65	4.04	2.42	11.61
**nNOS MP**	5	53.27	52.03	47.65	60.02	50.98	55.69	4.74	2.84	13.61
**nNOS OSP**	5	44.05	45.85	39.85	47.01	41.36	46.17	3.22	1.93	9.24
**nNOS ISP**	5	52.80	52.04	49.87	56.47	51.36	54.25	2.59	1.55	7.44
**VACHT MP**	5	13.78	14.37	10.38	15.62	13.85	14.69	2.01	1.20	5.77
**VACHT OSP**	5	22.27	22.36	18.45	26.20	19.58	24.75	3.30	1.97	9.47

**VACHT ISP**	5	30.80	30.36	23.52	35.52	29.85	34.75	4.80	2.87	13.78

Supplementary Table S2. Two-way analysis of variance (ANOVA) for the effect of experimental group, plexus, and interactions on number of nNOS-immunoreactive neurons.

**Effect**	**SS**	**df**	**MS**	**F**	**p-value**
Intercept	67893.10	1	67893.10	4134.301	0.000001
Experimental group	2979.91	2	1489.96	90.730	0.000001
Plexus	2043.85	2	1021.93	62.229	0.000001
Experimental group × Plexus	273.72	4	68.43	4.167	0.007103
Error	591.19	36	16.42		

Supplementary Table S3. Tukey HSD post-hoc test results for nNOS.

Plexus	Experimental group	C	C	C	G50	G50	G50	G500	G500	G500
MP	C		0.000143	0.110268	0.084572	0.000153	0.985389	0.000203	0.555785	0.000256
OSP	C	0.000143		0.034411	0.000140	0.999993	0.000252	0.000140	0.000140	0.000140
ISP	C	0.110268	0.034411		0.000155	0.078148	0.573501	0.000140	0.000650	0.000140
MP	G50	0.084572	0.000140	0.000155		0.000140	0.007167	0.254204	0.974394	0.346758
OSP	G50	0.000153	0.999993	0.078148	0.000140		0.000483	0.000140	0.000140	0.000140
ISP	G50	0.985389	0.000252	0.573501	0.007167	0.000483		0.000142	0.103953	0.000144
MP	G500	0.000203	0.000140	0.000140	0.254204	0.000140	0.000142		0.023681	1.000000
OSP	G500	0.555785	0.000140	0.000650	0.974394	0.000140	0.103953	0.023681		0.037740
ISP	G500	0.000256	0.000140	0.000140	0.346758	0.000140	0.000144	1.000000	0.037740	

Supplementary Table S4. Two-way analysis of variance (ANOVA) for the effect of experimental group, plexus, and interactions on number of VAChT-immunoreactive neurons.

**Effect**	**SS**	**df**	**MS**	**F**	**p-value**
Intercept	39485.24	1	39485.24	2700.010	0.000001
Experimental group	1472.47	2	736.23	50.344	0.000001
Plexus	1844.00	2	922.00	63.047	0.000001
Experimental group × Plexus	155.97	4	38.99	2.666	0.047896
Error	526.47	36	14.62		

Supplementary Table S5. Tukey HSD post-hoc test results for VAChT.

Plexus	Experimental group	C	C	C	G50	G50	G50	G500	G500	G500
MP	C		0.999416	0.000323	0.187073	0.107958	0.099579	0.000140	0.004486	0.998592
OSP	C	0.999416		0.000163	0.505694	0.345020	0.022855	0.000140	0.023294	1.000000
ISP	C	0.000323	0.000163		0.000140	0.000140	0.379635	0.000140	0.000140	0.000157
MP	G50	0.187073	0.505694	0.000140		0.999999	0.000186	0.000521	0.832968	0.556280
OSP	G50	0.107958	0.345020	0.000140	0.999999		0.000158	0.001009	0.935428	0.389155
ISP	G50	0.099579	0.022855	0.379635	0.000186	0.000158		0.000140	0.000140	0.018663
MP	G500	0.000140	0.000140	0.000140	0.000521	0.001009	0.000140		0.029824	0.000140
OSP	G500	0.004486	0.023294	0.000140	0.832968	0.935428	0.000140	0.029824		0.028434
ISP	G500	0.998592	1.000000	0.000157	0.556280	0.389155	0.018663	0.000140	0.028434	

Supplementary Table S6. Two-way analysis of variance (ANOVA) for the effect of experimental group, plexus, and interactions on number of CGRP-immunoreactive neurons.

**Effect**	**SS**	**df**	**MS**	**F**	**p-value**
Intercept	31400.17	1	31400.17	6921.682	0.000001
Experimental group	1145.31	2	572.65	126.233	0.000001
Plexus	845.52	2	422.76	93.191	0.000001
Experimental group × Plexus	8.13	4	2.03	0.448	0.773202
Error	163.31	36	4.54		

Supplementary Table S7. Tukey HSD post-hoc test results for CGRP.

Plexus	Experimental group	C	C	C	G50	G50	G50	G500	G500	G500
MP	C		0.000140	0.364589	0.147127	0.000722	0.998164	0.000140	0.871380	0.000140
OSP	C	0.000140		0.000152	0.000140	0.032743	0.000140	0.000140	0.000140	0.000140
ISP	C	0.364589	0.000152		0.000439	0.223956	0.092819	0.000140	0.015858	0.000140
MP	G50	0.147127	0.000140	0.000439		0.000140	0.495167	0.000366	0.903666	0.005925
OSP	G50	0.000722	0.032743	0.223956	0.000140		0.000197	0.000140	0.000144	0.000140
ISP	G50	0.998164	0.000140	0.092819	0.495167	0.000197		0.000140	0.998083	0.000147
MP	G500	0.000140	0.000140	0.000140	0.000366	0.000140	0.000140		0.000142	0.972371
OSP	G500	0.871380	0.000140	0.015858	0.903666	0.000144	0.998083	0.000142		0.000230
ISP	G500	0.000140	0.000140	0.000140	0.005925	0.000140	0.000147	0.972371	0.000230	

Supplementary Table S8. Two-way analysis of variance (ANOVA) for the effect of experimental group, plexus, and interactions on number of PACAP-immunoreactive neurons.

**Effect**	**SS**	**df**	**MS**	**F**	**p-value**
Intercept	15844.88	1	15844.88	1382.099	0.000001
Experimental group	2238.53	2	1119.26	97.630	0.000001
Plexus	1151.45	2	575.72	50.219	0.000001
Experimental group × Plexus	133.03	4	33.26	2.901	0.035259
Error	412.72	36	11.46		

Supplementary Table S9. Tukey HSD post-hoc test results for PACAP.

Plexus	Experimental group	C	C	C	G50	G50	G50	G500	G500	G500
MP	C		0.127299	0.005699	0.637571	0.726646	0.186094	0.000140	0.105341	0.179150
OSP	C	0.127299		0.938182	0.001068	0.960792	1.000000	0.000140	0.000166	0.000203
ISP	C	0.005699	0.938182		0.000155	0.310206	0.873711	0.000140	0.000140	0.000140
MP	G50	0.637571	0.001068	0.000155		0.023869	0.001773	0.000140	0.970373	0.994559
OSP	G50	0.726646	0.960792	0.310206	0.023869		0.986696	0.000140	0.001230	0.002479
ISP	G50	0.186094	1.000000	0.873711	0.001773	0.986696		0.000140	0.000190	0.000257
MP	G500	0.000140	0.000140	0.000140	0.000140	0.000140	0.000140		0.000140	0.000140
OSP	G500	0.105341	0.000166	0.000140	0.970373	0.001230	0.000190	0.000140		0.999999
ISP	G500	0.179150	0.000203	0.000140	0.994559	0.002479	0.000257	0.000140	0.999999	

Supplementary Table S10. Two-way analysis of variance (ANOVA) for the effect of experimental group, plexus, and interactions on number of CART-immunoreactive neurons.

**Effect**	**SS**	**df**	**MS**	**F**	**p-value**
Intercept	9352.524	1	9352.524	835.9644	0.000001
Experimental group	1243.915	2	621.958	55.5929	0.000001
Plexus	878.138	2	439.069	39.2456	0.000001
Experimental group × Plexus	16.194	4	4.048	0.3619	0.834076
Error	402.757	36	11.188		

Supplementary Table S11. Tukey HSD post-hoc test results for CART.

Plexus	Experimental group	C	C	C	G50	G50	G50	G500	G500	G500
MP	C		0.231068	0.001185	0.305729	0.993874	0.230273	0.000143	0.021682	0.994722
OSP	C	0.231068		0.467843	0.000578	0.035695	1.000000	0.000140	0.000147	0.037399
ISP	C	0.001185	0.467843		0.000140	0.000208	0.469024	0.000140	0.000140	0.000213
MP	G50	0.305729	0.000578	0.000140		0.819760	0.000575	0.007475	0.947439	0.810108
OSP	G50	0.993874	0.035695	0.000208	0.819760		0.035529	0.000203	0.158294	1.000000
ISP	G50	0.230273	1.000000	0.469024	0.000575	0.035529		0.000140	0.000147	0.037226
MP	G500	0.000143	0.000140	0.000140	0.007475	0.000203	0.000140		0.144973	0.000199
OSP	G500	0.021682	0.000147	0.000140	0.947439	0.158294	0.000147	0.144973		0.152388
ISP	G500	0.994722	0.037399	0.000213	0.810108	1.000000	0.037226	0.000199	0.152388	
